# Supplementary material for: Structural diversity and conservation among CRESS-DNA bacilladnaviruses revealed through cryo-EM and computational modelling
Source: Virol J. 2025 Dec 1;22:391. doi: 10.1186/s12985-025-03019-8 (PMC12667125; doi:10.1186/s12985-025-03019-8)
Supplement: Supplementary file 1 — Supplementary Material 1. [file 12985_2025_3019_MOESM1_ESM.docx]

Supplementary material for:

Structural diversity and conservation among CRESS-DNA bacilladnaviruses revealed through cryo-EM and computational modelling

L. Johanna Gebhard^1^, Yuji Tomaru^2^, Kenta Okamoto^1^, Anna Munke^1,3*^

1. Laboratory of Molecular Biophysics, Department of Cell and Molecular Biology, Uppsala University, Uppsala, Sweden
2. Fisheries Technology Institute, Japan Fisheries Research and Education Agency, Hatsukaichi, Hiroshima, Japan
3. Biochemistry & Structural Biology, Centre for Molecular Protein Science, Department of Chemistry, Lund University, Sweden

*Correspondence: [anna.munke@biochemistry.lu.se](mailto:anna.munke@biochemistry.lu.se)

Content:

Figure S1-S5

Table S1

Supplementary References


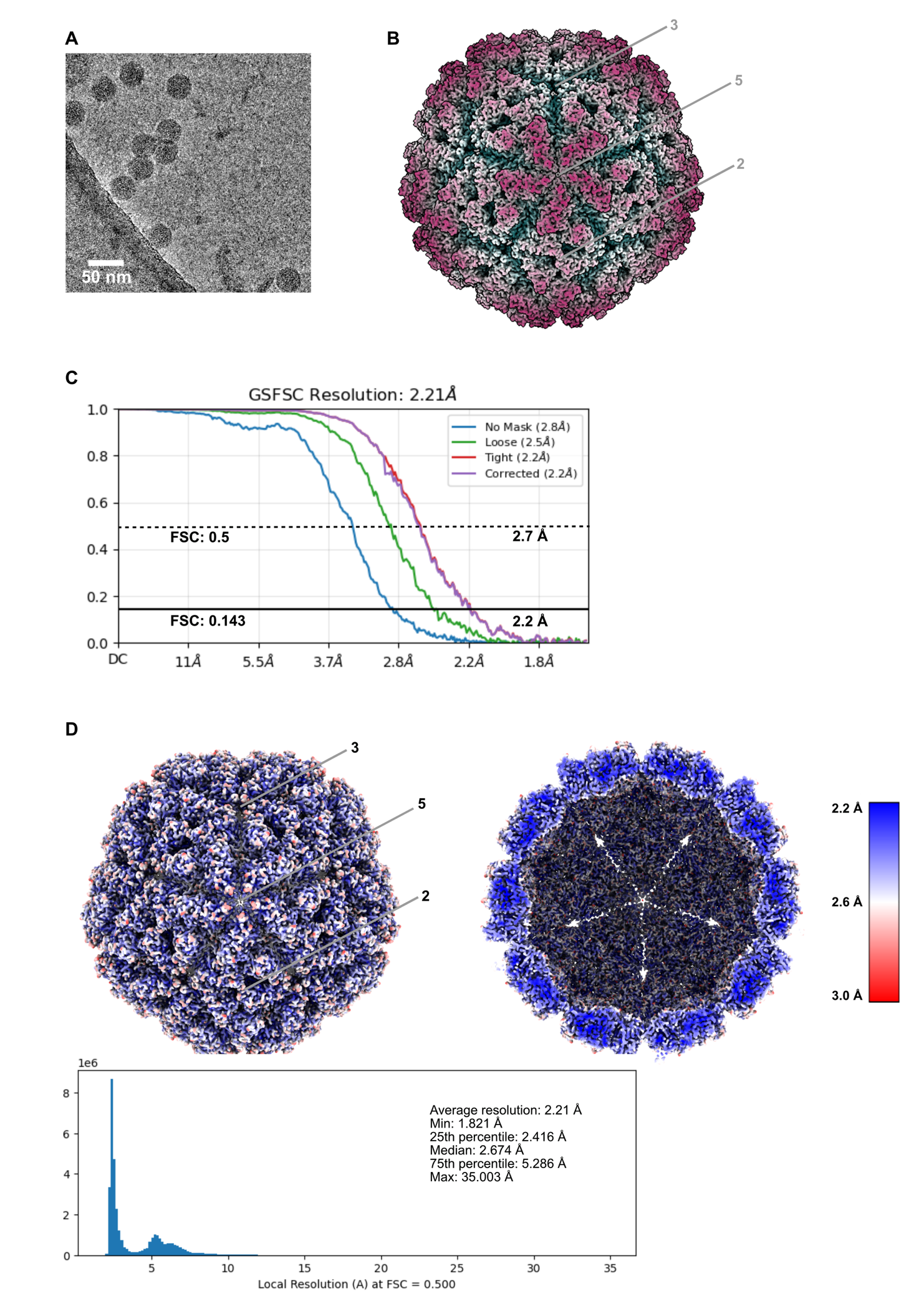
Supplementary Figures

**Figure S1:** Data collection and reconstruction of the ClorDNAV capsid. (A) Raw micrograph of ClorDNAV virions collected during cryo-EM imaging. (B) 3D reconstruction of the ClorDNAV capsid viewed down the 5-fold axis. The icosahedral 5-fold, 3-fold, and 2-fold symmetry axes are indicated as 5, 3, and 2, respectively. (C) FSC resolution curves generated by cryoSPARC, showing masked (blue), unmasked (green), FSC-mask tightened (red), and final corrected (purple) curves, estimated resolutions of ~2.7 Å at FSC = 0.5 (dotted line) and 2.2  Å at FSC = 0.143 (solid line). The resolution was determined to be 2.2 Å, corresponding to the spatial frequency at which the FSC curve drops below the 0.143 threshold [1,2]. (D) Local resolution map of the final capsid reconstruction, shown from the outside (left) and with the front half removed to show the interior (right). Color scale ranges from blue (2.2 Å) to white (2.6 Å) to red (3.0 Å). Histogram created by cryoSPARC shows voxel distribution by local resolution at FSC = 0.5.

**
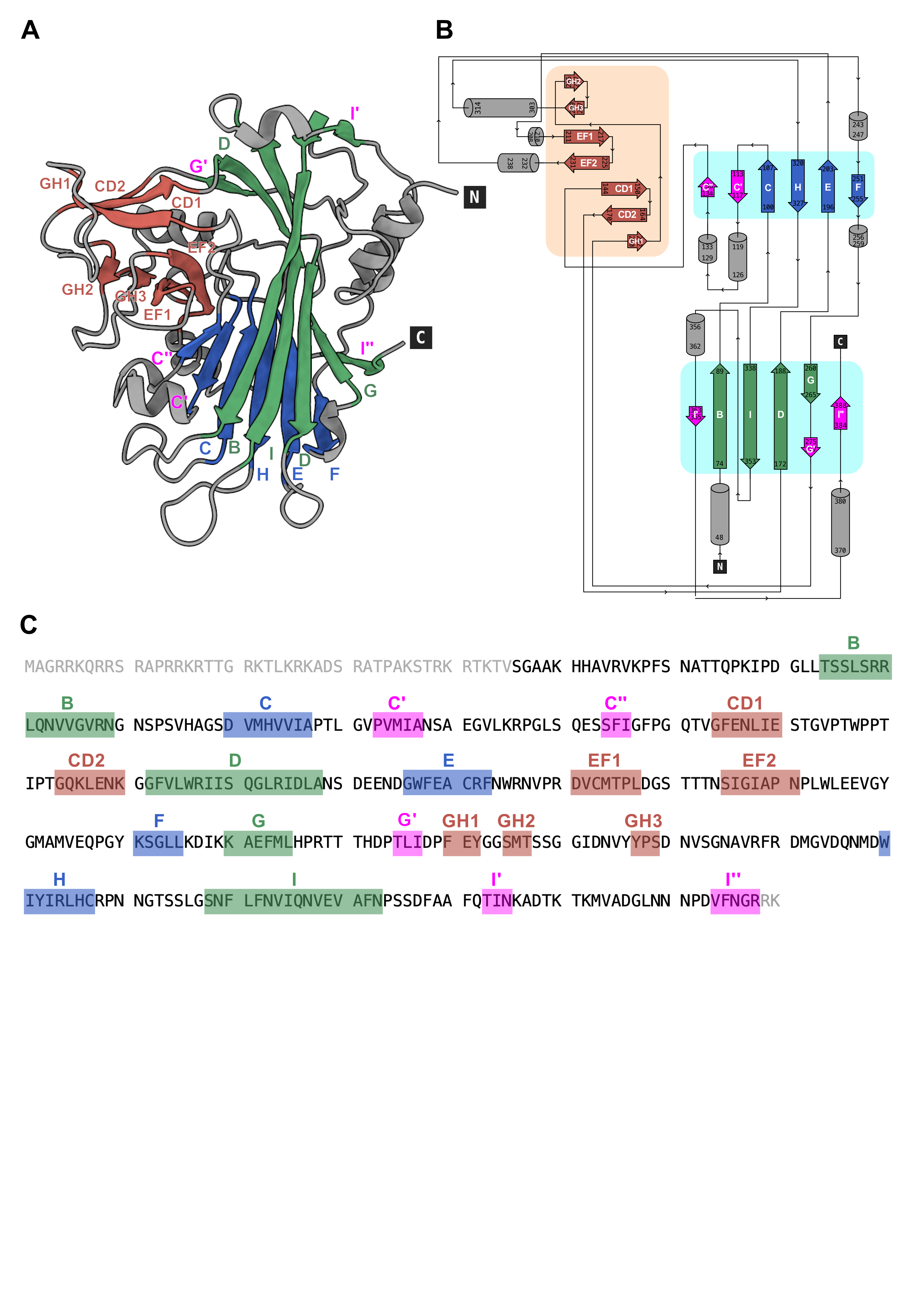
Figure S2:** Detailed structural topology of the ClorDNAV capsid protein. The β-strands are colored according to β-sheets, and named alphabetically (B to I) according to the conventional jelly-roll fold nomenclature in the green and blue β-sheets. The five additional β-strands (C’, C’’, G’, I’, and I’’) are highlighted in magenta (A) Secondary structure of ClorDNAV subunit A. (B) Schematic diagram showing the jelly-roll domain (light blue) and the projection domain (light yellow). (C) Amino acid sequence of subunit A, starting from residue 1 and divided into blocks of 10 residues. The respective β-strand is marked above the sequence. The first 45 residues, which could not be modelled, are shown in grey and are enriched in positively charged residues (21 total).


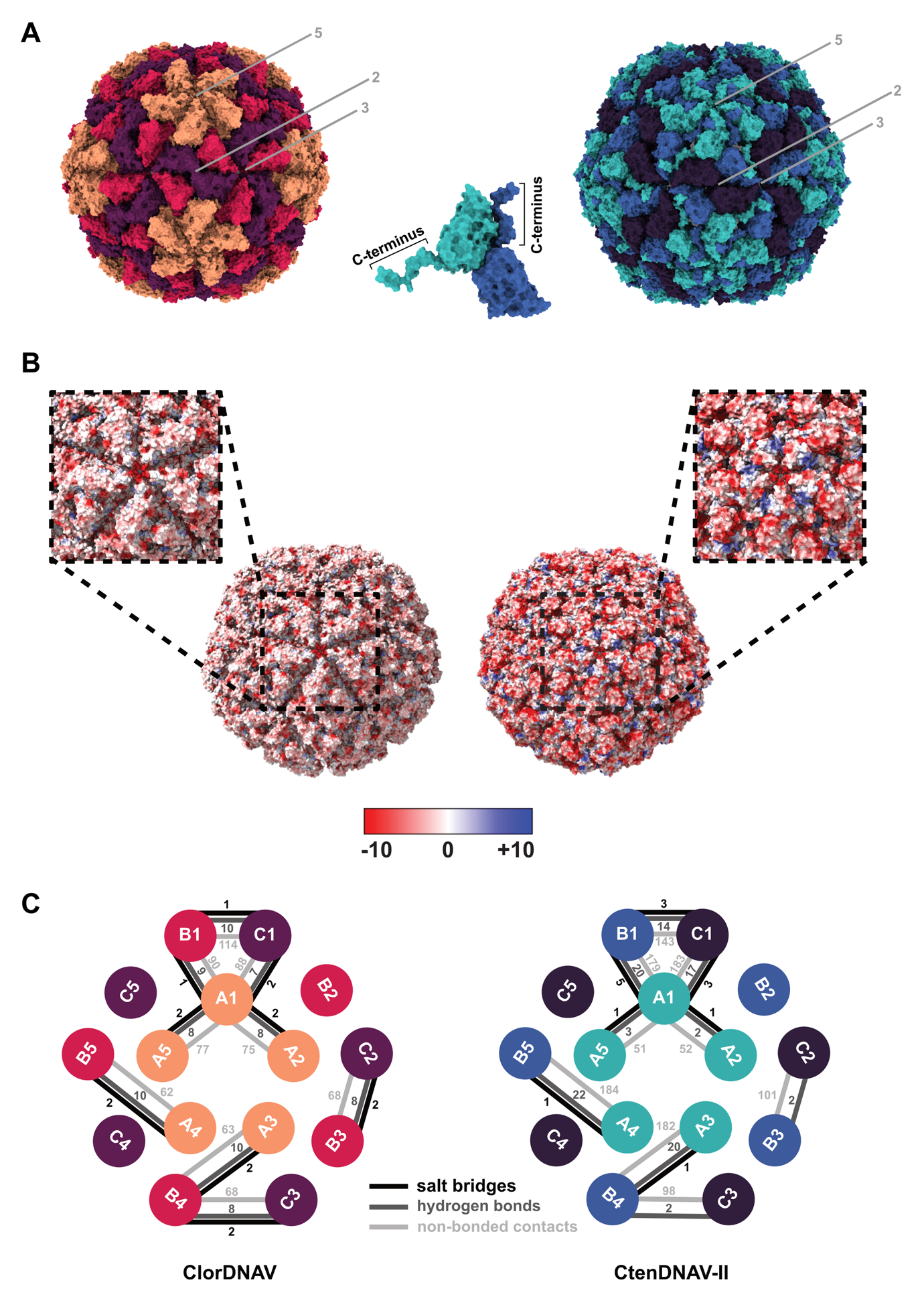
**Figure S3:** Biochemical characterization of the ClorDNAV (left) and CtenDNAV-II (right) capsid [3]. (A) Capsid models colored by subunit. A close-up view of one A and B subunit from CtenDNAV-II is shown to highlight the surface-exposed C-termini. (B) Capsid models colored according to their coulombic electrostatic potential, from red (negative) to white (neutral) to blue (positive). Insets show close-ups of the 5-fold symmetry axis. Analysis was performed with ChimeraX v1.9 [4]. (C) Comparison of subunit interactions based on PDBsum analysis [5], showing the number of predicted salt bridges (black), hydrogen bonds (dark grey), and non-bonded contacts (light grey).

**
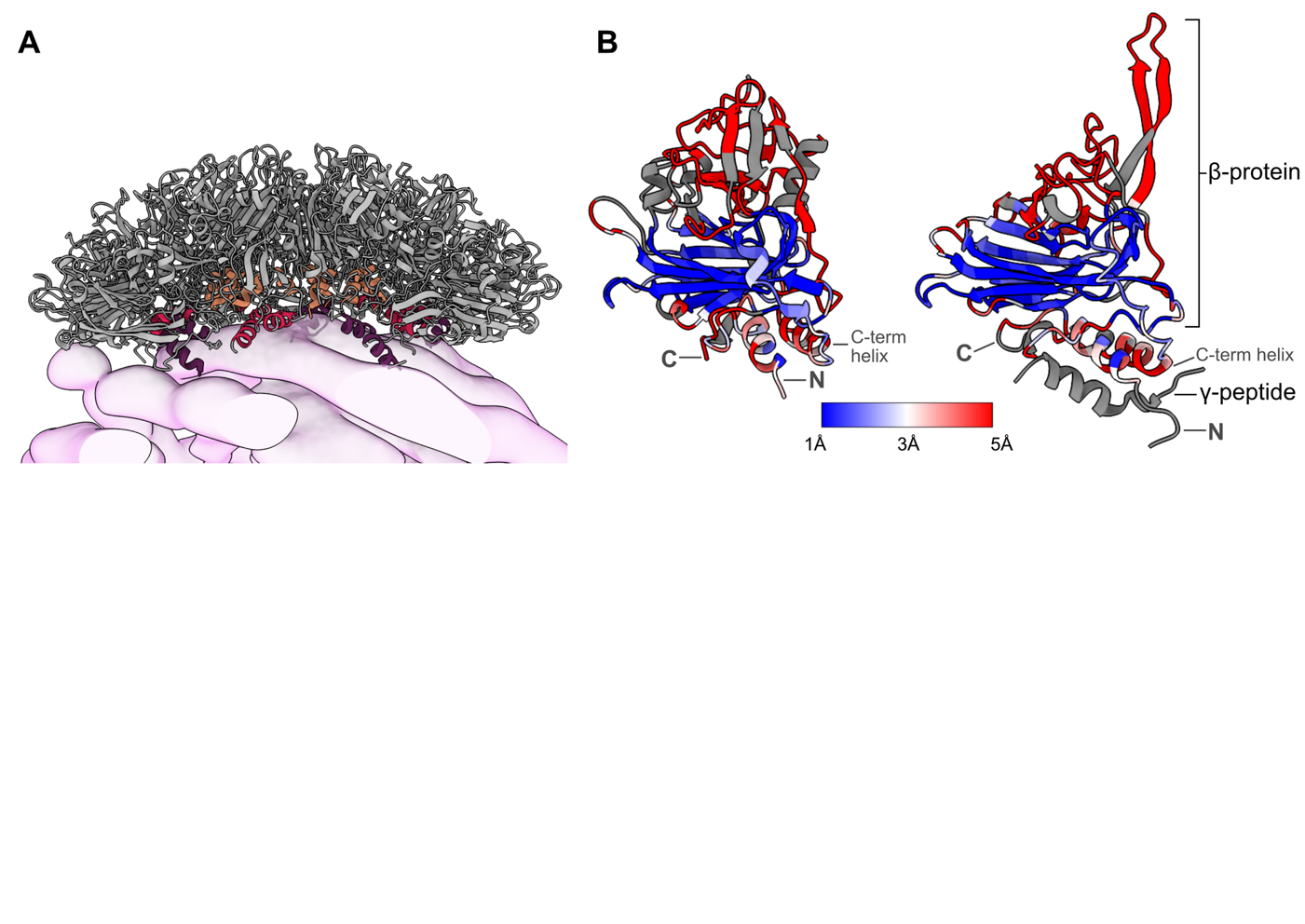
**

**Figure S4:** The N and C-terminal α-helices in the ClorDNAV CP are orientated towards the capsid interior (A) Terminal α-helices shown for a ClorDNAV 15-mer, colored by subunit (A: orange, B: red, C: purple). Helices are positioned towards the capsid interior and may interact with the outer genome layer (light pink). (B) Comparison of ClorDNAV subunit A (left) to a Nodamura Virus CP subunit (PDB: 1NOV, right), colored by RMSD from low (blue) to high (red) distance; missing regions are shown in grey. The 1NOV subunit is composed of the β-protein and the γ-peptide [6].


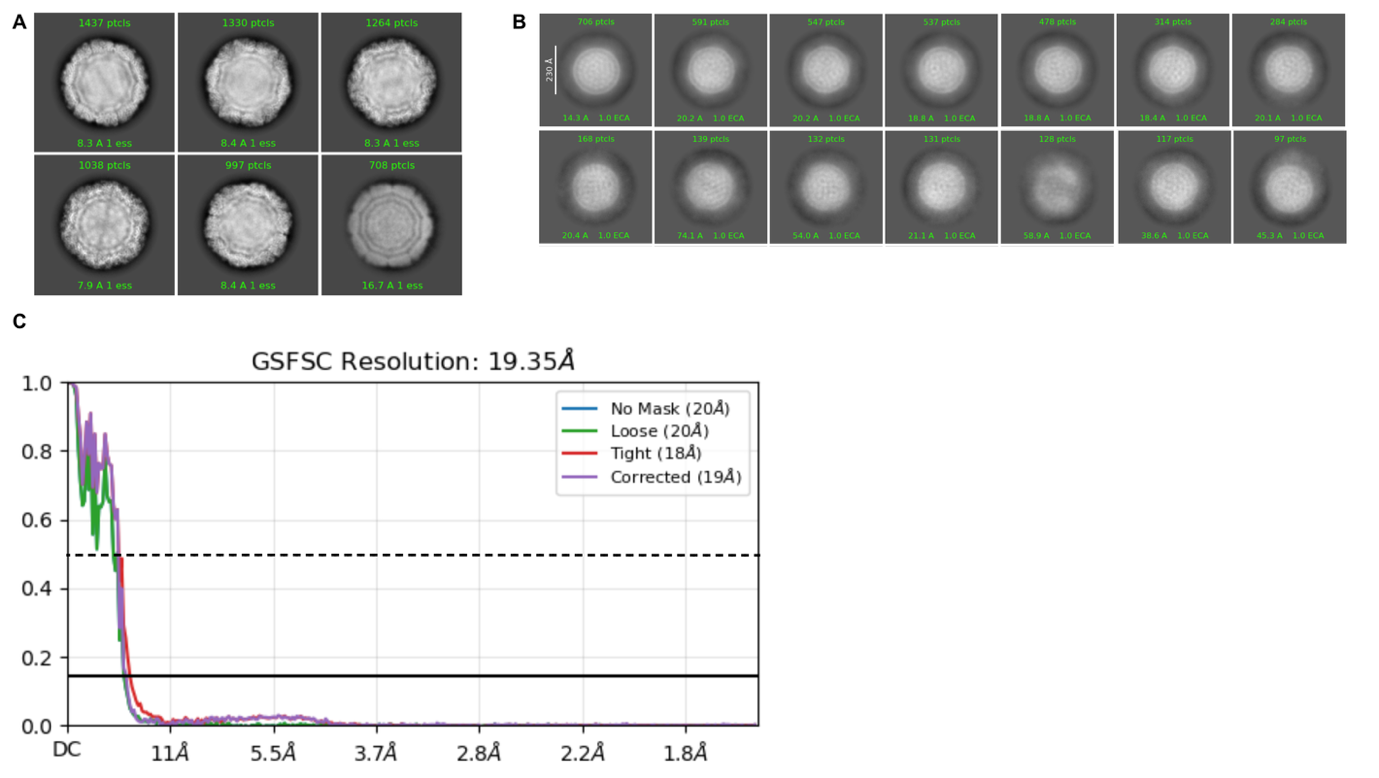
**Figure S5:** Data processing of the outer genome layer of ClorDNAV. (A) Representative 2D classes of ClorDNAV particles showing internal structural features. (B) 2D classes after signal subtraction of the capsid, highlighting genome-associated density. (C) FSC curves of the outer genome layer reconstruction, with estimated resolutions of ~21 Å at FSC = 0.5 (dotted line) and ~19 Å at FSC = 0.143 (solid line).

Supplementary Tables

**Table S1:** Summary of data collection and processing parameters, along with validation statistics for the model, the data, and model-to-data agreement generated by PHENIX [7–9].

|  | ClorDNAV capsid EMD-54550 | Outer genome layer  EMD-54548 |
| --- | --- | --- |
| **Data collection and processing** | | |
| Magnification | 105,000 | |
| Voltage (kV) | 300 | |
| Defocus range (μm) | -0.4 to -1.2 | |
| Microscope | Titan Krios G2 | |
| Camera | K3 BioQuantum | |
| Total electron dose (e^-^/Å^2^) | 45.156 | |
| Pixel size (Å) | 0.825 | |
| Number of micrographs | 9,352 | |
| Final particle number | 8,145 | 3,117 |
| Symmetry imposed | I | C1 |
| Map resolution (Å) (FSC=0.143) | 2.21 | 19.35 |
| Map resolution (Å) (FSC=0.5) | ~2.7 | ~21 |
| Map resolution range (Å) | 1.82-35 | NA |
| Map sharpening B-factor (Å^2^) | -61.0 |  |
| **Model** | | |
| PDB | 9S3S |  |
| Model Composition  Chains  Non-hydrogen atoms  Protein residues  Ligands | 3  7,681  1,028  0 |  |
| R.m.s. deviations  Bond lengths (Å)  Bond angles (°) | 0.003  0.546 |  |
| Validation  MolProbity score  Clashscore  Poor rotamers (%)  Cβ outliers (%)  CaBLAM outliers (%)  Cis proline / general (%)  Twisted proline / general (%) | 0.84  1.22  0.00  NA  1.48  8.3/0.0  0.0/0.0 |  |
| Rama-Z  Whole (N = 1022)  Helix (N = 136)  Sheet (N = 298)  Loop (N = 588) | 0.43 (0.27)  0.57 (0.44)  0.35 (0.30)  0.32 (0.27) |  |
| Ramachandran plot (%)  Favored (%)  Allowed (%)  Outliers (%) | 98.24  1.76  0.00 |  |
| B-factors (Å^2^) (min/max/mean) | 17.83/82.66/34.59 |  |
| **Data** | | |
| d99 masked (full/half1/half2) | 2.35/1.69/1.69 |  |
| d99 unmasked (full/half1/half2) | 2.28/1.67/1.67 |  |
| FSC (model) = 0 (masked/unmasked) | 2.11/2.18 |  |
| FSC (model) = 0.143 (masked/unmasked) | 2.18/2.21 |  |
| FSC (model) = 0.5 (masked/unmasked) | 2.31/2.79 |  |
| **Model vs Data** | | |
| CC (mask) | 0.91 |  |
| CC (box) | 0.47 |  |
| CC (peaks) | 0.33 |  |
| CC (volume) | 0.89 |  |
| EMRinger score | 6.68 |  |

Supplementary References

1. Henderson R, Sali A, Baker ML, Carragher B, Devkota B, Downing KH, et al. Outcome of the First Electron Microscopy Validation Task Force Meeting. Structure. 2012;20:205–14.

2. Scheres SHW, Chen S. Prevention of overfitting in cryo-EM structure determination. Nature Methods. 2012;9:853–4.

3. Munke A, Kimura K, Tomaru Y, Wang H, Yoshida K, Mito S, et al. Primordial Capsid and Spooled ssDNA Genome Structures Unravel Ancestral Events of Eukaryotic Viruses. Mbio. 2022;13:e00156-22.

4. Pettersen EF, Goddard TD, Huang CC, Meng EC, Couch GS, Croll TI, et al. UCSF ChimeraX: Structure visualization for researchers, educators, and developers. Protein Science. 2021;30:70–82.

5. Laskowski RA, Jabłońska J, Pravda L, Vařeková RS, Thornton JM. PDBsum: Structural summaries of PDB entries. Protein Science. 2018;27:129–34.

6. Zlotnick A, Natarajan P, Munshi S, Johnson JE. Resolution of Space-Group Ambiguity and Structure Determination of Nodamura Virus to 3.3 Å resolution from Pseudo-ıt R32 (Monoclinic) Crystals. Acta Crystallographica Section D. 1997;53:738–46.

7. Adams PD, Afonine PV, Bunkóczi G, Chen VB, Davis IW, Echols N, et al. PHENIX: a comprehensive Python-based system for macromolecular structure solution. Acta Crystallographica Section D. 2010;66:213–21.

8. Afonine PV, Klaholz BP, Moriarty NW, Poon BK, Sobolev OV, Terwilliger TC, et al. New tools for the analysis and validation of cryo-EM maps and atomic models. Acta Crystallographica Section D. 2018;74:814–40.

9. Williams CJ, Headd JJ, Moriarty NW, Prisant MG, Videau LL, Deis LN, et al. MolProbity: More and better reference data for improved all-atom structure validation. Protein Science. 2018;27:293–315.
